# Activating the interleukin-6-Gp130-STAT3 pathway ameliorates ventricular electrical stability in myocardial infarction rats by modulating neurotransmitters in the paraventricular nucleus

**DOI:** 10.1186/s12872-020-01363-x

**Published:** 2020-02-05

**Authors:** Meng Gao, Dechun Yin, Jugang Chen, Xiufen Qu

**Affiliations:** Department of Cardiology, the First Affiliated Hospital of Harbin Medical University, Harbin, 150001 China

**Keywords:** Hypothalamic paraventricular nucleus, Interleukin-6, Glycoprotein 130, STAT3, Sympathetic activity, Cardiac electrophysiological activity

## Abstract

**Background:**

Malignant ventricular arrhythmia (VA) is the most common cause of death associated with acute myocardial infarction (MI). Recent studies have revealed direct involvement of the paraventricular nucleus (PVN) in the occurrence of VA. However, the underlying mechanisms remain incompletely understood. In this study, we investigated changes in the interleukin-6 (IL-6)-glycoprotein 130-signal transducer and activator of transcription 3 (STAT3) pathway in the PVN during acute MI and the effects of this pathway on ventricular stability.

**Methods:**

Rats were divided into a control group, a MI group, a PVN-injected anti-IL-6 antibody group and a PVN-injected SC144 group to observe how IL-6 and its downstream glycoprotein 130-STAT3 pathway in the PVN affect ventricular stability. The left anterior descending coronary artery was ligated to induce MI. After that, an anti-IL-6 antibody and SC144 were injected into the PVNs of rats. All data are expressed as the mean ± SE and were analysed by ANOVA with a post hoc LSD test. *p* < 0.05 was considered to indicate statistical significance.

**Results:**

After MI, the concentration of the inflammatory factor IL-6 increased, and its downstream glycoprotein 130-STAT3 pathway was activated in the PVN. After injection of MI rat PVNs with the anti-IL-6 antibody or glycoprotein 130 inhibitor (SC144), glutamate levels increased and γ-aminobutyric acid (GABA) levels decreased in the PVN. Plasma norepinephrine concentrations also increased after treatment, which increased the vulnerability to VA.

**Conclusions:**

In summary, IL-6 in the PVN exerts a protective effect in MI rats, and the glycoprotein 130-STAT3 pathway plays a key role in this process. We anticipate that our findings will provide new ideas for the prevention and treatment of arrhythmia after MI.

## Background

Acute myocardial infarction (MI) is a condition of myocardial necrosis caused by acute, persistent ischaemia and hypoxia in the coronary arteries [1]. There are some complications of MI, including heart failure, arrhythmia, heart rupture, pericarditis, papillary muscle rupture and others. Arrhythmia occurs in most MI patients and most commonly occurs within 24 h [2]. Furthermore, lethal ventricular arrhythmia (VA) is the most common cause of death among patients with acute MI. It is well known that autonomic imbalance, especially excessive activation of sympathetic nerves (called a sympathetic storm), plays the most important role in promoting the occurrence of arrhythmia. In recent years, there have been many reports on the mechanisms by which peripheral autonomic nerves, such as local cardiac nerves, renal sympathetic nerves, and star ganglions, regulate arrhythmia [3, 4]. However, the mechanism by which the central nervous system (CNS) affects VA remains unclear.

Lampert et al. have demonstrated that ventricular tachycardia and ventricular fibrillation (VF) can be induced by psychological stress, sudden changes in mental state, brain trauma, and elevated intracranial pressure [5]. Davis et al. have demonstrated that brain tissue regions and nuclei from the medulla to the cerebral cortex play important roles in the development of arrhythmia and revealed that there are complex and variable interconnections among these areas [6]. Stimulation of different brain regions and nerve nuclei can lead to different types of arrhythmia. Among these regions, the paraventricular nucleus (PVN) is the main area of sympathetic preganglionic neuron accumulation and innervates other autonomic nuclei, including the midbrain periaqueductal grey region, the parabrachial region, the rostral ventrolateral medulla, the solitary tract nucleus, the dorsal vagal nucleus and the nucleus ambiguus. Moreover, the PVN is an important integrative site within the brain composed of magnocellular and parvocellular neurons. Parvocellular neurons project to other sites within the CNS, including regions that are important for autonomic control [7, 8]. However, the exact mechanism by which the PVN affects arrhythmia remains unclear and needs further investigation. Changes in neurochemical factors, such as reactive oxygen species and inflammatory cytokines, in the hypothalamic PVN during MI may be important factors in the increase in sympathetic nerve sensitivity that occurs during MI. Kang et al. have shown that microinjection of pro-inflammatory cytokine inhibitors into the CNS can alleviate the symptoms of MI and that the effects of central administration are significantly better than those of peripheral administration [9, 10]. Neurotransmitters play important roles in this process. For example, glutamate is enhanced and γ-aminobutyric acid (GABA) declines in the PVN during MI, thereby affecting sympathetic overactivation and further affecting heart function [11]. Glutamate, one of the most important excitatory amino acids in the CNS, regulates sympathetic nerve activity and cardiovascular function through N-methyl-D-aspartic acid (NMDA) receptors. Stimulation of NMDA receptors in the PVN can increase sympathetic discharge. GABA is the main inhibitory neurotransmitter in the PVN of the hypothalamus. Injecting GABA into the PVN of the hypothalamus can reduce heart rate and attenuate arrhythmia. GAD67 is a rate-limiting enzyme of GABA and a marker for GABAergic neurons, and its distribution is parallel to that of GABA.

In contrast to other inflammatory factors, interleukin-6 (IL-6) is a pleiotropic regulator that has multiple functions, not only exerting pro-inflammatory effects but also affecting tissue regeneration, metabolism and other processes. IL-6 is upregulated significantly during acute injury and plays key roles in mediating the acute-phase response. IL-6 has two kinds of receptors, a membrane-bound receptor and a soluble receptor, both of which can bind to Glycoprotein 130 (Gp130). After dimerization, intracellular signalling occurs through IL-6 classic signalling and trans-signalling pathways. Interestingly, these two pathways strongly differ in their biological influences. While classic signalling is primarily associated with protection, promoting tissue regeneration and maintaining physiological homeostasis, trans-signalling has deleterious effects [12]. With regard to the CNS, Suzuki et al. demonstrated that IL-6 plays a protective role in the early stage of brain injury. Intracerebroventricular injection of rhIL-6 dramatically reduces ischaemic brain damage measured 24 h after middle cerebral artery occlusion [13–15].

Gp130 is a receptor of IL-6 and is the main signalling molecule for intracellular signal transduction. Currently, three signalling pathways are known to be associated with Gp130: the JAK-signal transducer and activator of transcription (STAT) pathway, the EKA pathway, and the PI3K/Akt pathway. The most prominent proteins recruited to Gp130 are the STAT family transcription factors STAT3 and (to a certain extent) STAT1. Furthermore, it is currently well accepted that STAT3 and (to a much lesser extent) STAT1 are activated by IL-6. Binding to IL-6 causes phosphorylation of Gp130 and then activates the cytoplasmic region [16, 17]. Gp130 phosphorylation exposes a STAT3 binding site to induce STAT3 phosphorylation and then enters the nucleus to initiate transcription. Habecker et al. confirmed that Gp130 mediates the conversion of peripheral sympathetic neurons to cholinergic neurons after MI [18]. The sympathetic co-release of acetylcholine (Ach) and norepinephrine (NE) impairs adaptation to high heart rates and increases arrhythmia susceptibility. In the CNS, the Gp130 pathway promotes the differentiation and growth of nerves [17, 19]. However, the effect of Gp130 on neurotransmitter conversion in the PVN has not been studied.

The aim of this study is to investigate whether IL-6 in the hypothalamic PVN exerts a protective effect against the incidence of VA after MI and whether the Gp130-STAT3 pathway plays a key role in this process.

## Methods

### Animals

Adult male Sprague-Dawley rats (200–250 g) were purchased from the Animal Experimental Center of the Second Affiliated Hospital of Harbin Medical University. The rats were housed at a density of 8 rats per cage with 12 h of light and freely available food and water at a temperature 23 ± 2 °C and a relative humidity of 40–50%.

### Coronary ligation and paraventricular nucleus injection (PNI) [20, 21]

The rats underwent sterile surgery under anaesthesia (Ulatan, concentration:20%, 150 mg/kg, intraperitoneal [i.p.]) for induction of MI by ligation of the left anterior descending coronary artery (MI group) or the same surgery without ligation of the vessel (sham group). The PVN in each rat was injected with artificial cerebrospinal fluid (ACSF; given to sham rats and MI rats), an anti-IL-6 antibody or a Gp130 antagonist (SC144) according to the rat stereotaxic atlas coordinates, and each group included 12 rats (*n* = 12) [23-24].

### Cardiac electrophysiological studies

After the surgery, we recorded the arrhythmia occurrence in rats within 24 h using a single-lead dynamic electrocardiogram (Good Friend, Shenzhen, China). The rats were anaesthetized by i.p. injection of Ulatan (150 mg/kg), and a second thoracotomy was carried out to perform an open-chest electrophysiological study for assessment of endpoints including the VF threshold (VFT) and VF inducibility. A 1.9 F electrophysiological catheter (Scisense, Canada) was placed on the left ventricle, and eight poles recorded electrocardiograms with an Electrophysiology Lab Amplifier (GY-6000, Huanan Medical Science and Technology, Henan, China). To determine the VFT, the minimum voltage to induce sustained VF, 60 ms S1-S1 stimuli were repeatedly applied to the left ventricular apex, and the stimulus intensity was increased by 0.5 V each time until VF was induced. Ten bursts of ventricle pacing (25 Hz) lasting for 10 s each were used to assess the inducibility of VF. VF was defined as > 1000 ms of irregular VA.

### Methods of animal euthanasia and tissue collection

The animals were sacrificed by rapid excision of the heart to confirm permanent cessation of the circulation under anaesthesia (Ulatan, concentration:20%, 150 mg/kg i.p.). Then the rats were decapitated to get the whole brain. For western blotting, brain tissue was quickly extracted in a low-temperature environment, and Palkovits’s microdissection procedure was used to isolate the PVN. For immunohistochemistry, 4% paraformaldehyde was inserted into the left ventricle and the ascending aorta to fix the brain tissue, and then the rats were decapitated to obtain the brains.

### Immunohistochemistry

After the brain tissue was embedded in paraffin, the part between the optic chiasm and mammillary body was resected in the rostro-caudal direction. The tissue was serially sectioned on a paraffin slicer, and sections that were approximately 1.50 mm from the bregma were obtained [25, 26]. After dewaxing the slices, 3% H_2_O_2_ was used to block endogenous peroxidase activity, and 0.01 M citric acid was used to retrieve the antigens prior to antibody incubation. Then, the slices were incubated with primary antibodies overnight at 4 °C [27, 28]. The sections were immunohistochemically labelled to identify IL-6 (Bioss, China, 1:100), Gp130 (Santa Cruz, America, 1:20), pSTAT3 (Bioss, China, 1:50), NMDA receptors (Bioss, China, 1:50), and GAD67 (Abcam, England, 1:2000) and then incubated with secondary antibodies (anti-mouse for Gp130 and GAD67; anti-rabbit for NMDA receptors, pSTAT3 and IL-6) for 20 min at room temperature. For each rat, the positive neurons within the bilateral borders of the PVN were manually counted in three consecutive sections, and the average value is reported.

### Western blot analysis

The tissue was homogenized in RIPA buffer containing a protease inhibitor cocktail (Beyotime Biotechnology). A BCA protein assay (Beyotime Biotechnology) was used to determine the protein concentrations. Equal amounts of protein were separated by SDS-PAGE and then transferred electrophoretically to polyvinylidene fluoride membranes (Bio-Rad) [29, 30]. The membranes were incubated with the following primary antibodies for 2 h at room temperature: IL-6 (1:1000, Abcam, England), Gp130 (1:1000, Santa, America), pSTAT3 (1:1000, Bioss, China), and NMDA receptors (1:1000, Bioss, China). The membranes were then incubated with GAPDH (1:1000, Solarbio, China), goat anti-mouse IgG (Bioss, China, Gp130, 1:1000 and GAD67, 1:1000) or goat anti-rabbit IgG (Bioss, China, pSTAT3, 1:1000, NMDA receptors, 1:3000 and IL-6, 1:1000) secondary antibodies for 2 h at room temperature. Finally, the membranes were placed in a gel imaging analysis system for exposure and analysis (AlphaView FluorChem FC3).

### Measurement of glutamate and GABA in PVN tissues

Brain tissue was separated as previously described. Perchloric acid (0.1 mol/L, Sigma) was added to the brain tissue. Then, the tissue was dissolved on an ice pack or crushed ice, fully crushed and homogenized, and sonicated for 5 min. Finally, the samples were centrifuged at 12,000 rpm for 10 min at 4 °C. The supernatant was aspirated, diluted and filtered through a filtration membrane. The concentrations of glutamate and GABA were measured using a liquid chromatograph mass spectrometer (Singapore, Xevo).

### Measurement of circulating catecholamine levels

Arterial blood was drawn from the left heart chamber and centrifuged at 3000 rpm for 15 min at 4 °C. The supernatant was obtained and stored in a freezer at − 80 °C. An ELISA kit purchased from Bioss was used to measure NE levels. The standards were diluted and loaded for a total well volume of 50 μl. The standard concentrations were 120 ng/L, 80 ng/L, 40 ng/L, 20 ng/L, and 10 ng/L. The samples to be tested on the enzyme-labelled plate were first diluted; 40 μl and then 10 μl of each sample was added. Fifty microliters of enzyme labelling reagent were added per well. After sealing it with a sealing film, the plate was incubated at 37 °C for 30 min. The plate was zeroed with blank wells, and the absorbance of each well was measured in sequence at 450 nm and 630 nm wavelengths. The concentration in each sample was calculated based on the absorbance.

### Statistical analysis

All analyses were carried out with Statistical Product and Service Solutions 17.0 (SPSS Inc., Chicago, II, USA). Normality testing was performed to evaluate whether the data conforms the normal distribution prior to the analysis. All data were expressed as the mean ± SEM, data which fit the normal distribution were analysed by ANOVA followed by a post-hoc LSD test. Statistical significance was accepted at *p* < 0.05 for all analyses.

## Results

### Cardiac electrophysiological study

**Fig. 1:**
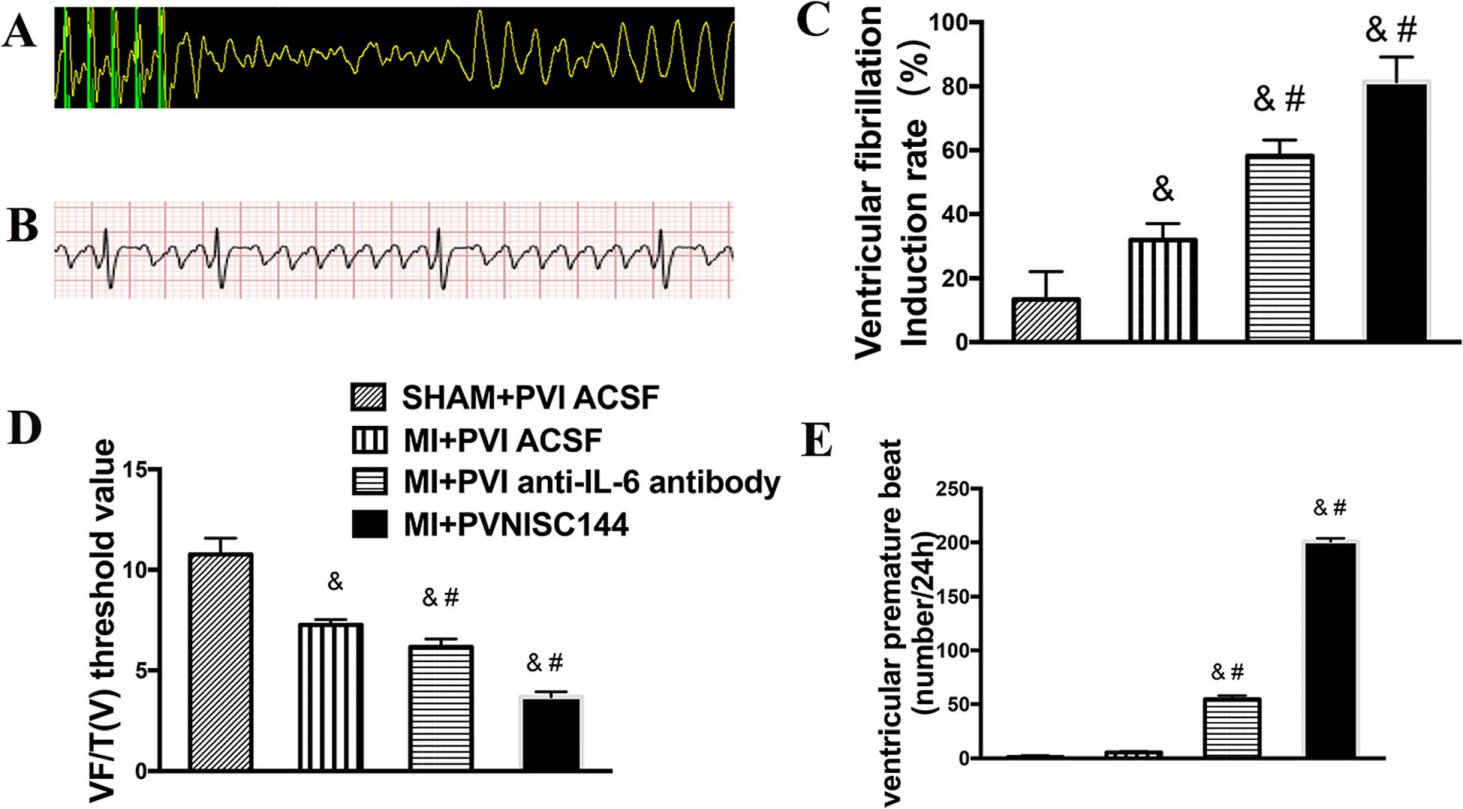
Effects of PVN infusion of an anti-IL-6-antibody or SC144 on ventricular electrophysiological activity in MI rats. **a** Recordings of typical ventricular arrhythmia induced by programmed electrical stimulation. **b** Recording of ventricular premature beats within 24 h by small-animal dynamic electrocardiography. **c** Quantitative analysis of induced ventricular fibrillation. **d** Quantitative analysis of the ventricular fibrillation threshold. **e** Quantitative analysis of premature ventricular beats over 24 h. The values are the means±SEs. ^&^*p* < 0.05 vs sham rats, ^#^*p* < 0.05 vs MI rats. Abbreviations: PVN, paraventricular nucleus; IL-6, interleukin-6; MI, myocardial infarction

Figure 1a shows a typical graph of VF induced by an electrophysiological catheter. Figure 1b displays a ventricular premature beat recorded by dynamic electrocardiography. As shown in Fig. 1c-e, MI rats showed a 4-fold greater incidence of spontaneous VA (5.5 ± 0.8 vs 1.3 ± 0.6) (*p* < 0.01) compared with sham rats. The incidence of spontaneous VA (54.67 ± 5.59 and 200.8 ± 19.12) in anti-IL-6 antibody-treated and SC144-treated PNI rats was significantly higher than that in sham rats and MI rats (*p* < 0.0001). To further study the changes in cardiac electrophysiology, we measured the catheter-induced VF and VFT in each group of rats. The induction rates of VF in MI rats were over 50% higher than those in sham rats (31.83 ± 3.43% vs 13.33 ± 1.41%) (*p* < 0.01), while anti-IL-6 antibody-treated rats and SC144-treated PNI rats had about 2-fold and 3-fold induction rates of VF (58.17 ± 5.74% and 81.33 ± 6.01%, respectively) when compared with MI rats (*p* < 0.0001). The VFT of MI rats (7.25 ± 0.63 V), in comparison with sham rats (10.75 ± 1.20 V), showed a 25% decrease (*p* < 0.0001), and the VFT of PNI anti-IL-6 antibody-treated PNI rats and SC144-treated PNI rats (6.16 ± 0.60 V and 3.66 ± 0.45 V) lessened 15 and 40% than in MI rats (*p* < 0.05).

### IL-6, Gp130 and pSTAT3 expression in the PVN

**Fig. 2:**
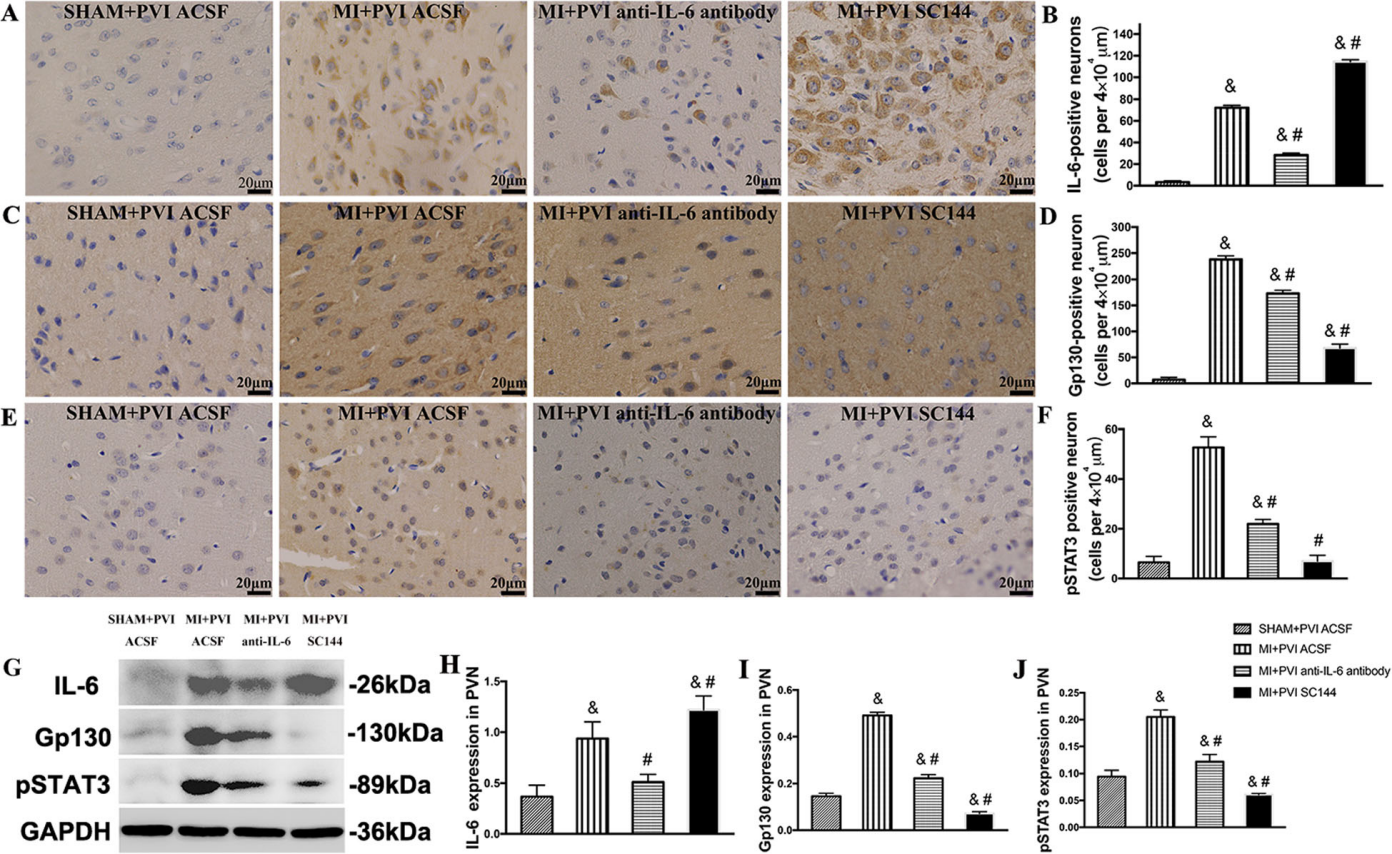
Effects of PVN infusion of an anti-IL-6 antibody or SC144 on IL-6, Gp130 and pSTAT3 levels within the MI rats. **a** Representative image of IL-6 immunohistochemical staining. **b** Densitometric analysis of IL-6 staining. **c** Representative image of Gp130 immunohistochemical staining. **d** Densitometric analysis of Gp130 staining. **e** Representative image of pSTAT3 immunohistochemical staining. **f** Densitometric analysis of pSTAT3 staining. **g** Representative immunoblot image of IL-6, Gp130 and pSTAT3 levels. **h-j** Densitometric analysis of the protein expression of IL-6, Gp130 and pSTAT3 (*n* = 4). The values are the means ± SEs. ^&^*p* < 0.05 vs sham rats, ^#^*p* < 0.05 vs MI rats. Abbreviations: PVN, paraventricular nucleus; IL-6, interleukin-6; MI, myocardial infarction; Gp130, glycoprotein 130

The immune system was activated when the left anterior descending coronary artery was ligated. The immunohistochemical images in Fig. 2a show the expression of IL-6 in the four groups and those in Fig. 2c illustrate the expression of Gp130 in the four groups. The expression of pSTAT3 in the four groups are displayed in Fig. 2e. Figure 2b, d and f show the densitometric analysis results for IL-6, Gp130 and pSTAT3, respectively. Figure 2g shows a representative immunoblot image of IL-6, Gp130 and pSTAT3 levels. From these images, we observed that compared to sham rats PVNs, MI rat PVNs had significantly higher IL-6 concentrations (*p* < 0.0001). In addition, the elevations in IL-6 activated the Gp130 receptor (*p* < 0.0001) and its downstream mediator pSTAT3 (*p* < 0.01). With the reduction in IL-6 content upon injection of the anti-IL-6 antibody, Gp130 and pSTAT3 activation was blunted (*p* < 0.05). Gp130 and STAT3 activation was also blunted conspicuously by SC144, which could bind to Gp130 and eventually abrogate STAT3 phosphorylation and nuclear translocation. Figure 2h-j show the densitometric analysis results for the protein expression of IL-6, Gp130 and pSTAT3.

### NMDA and GAD67 expression in the PVN

**Fig. 3:**
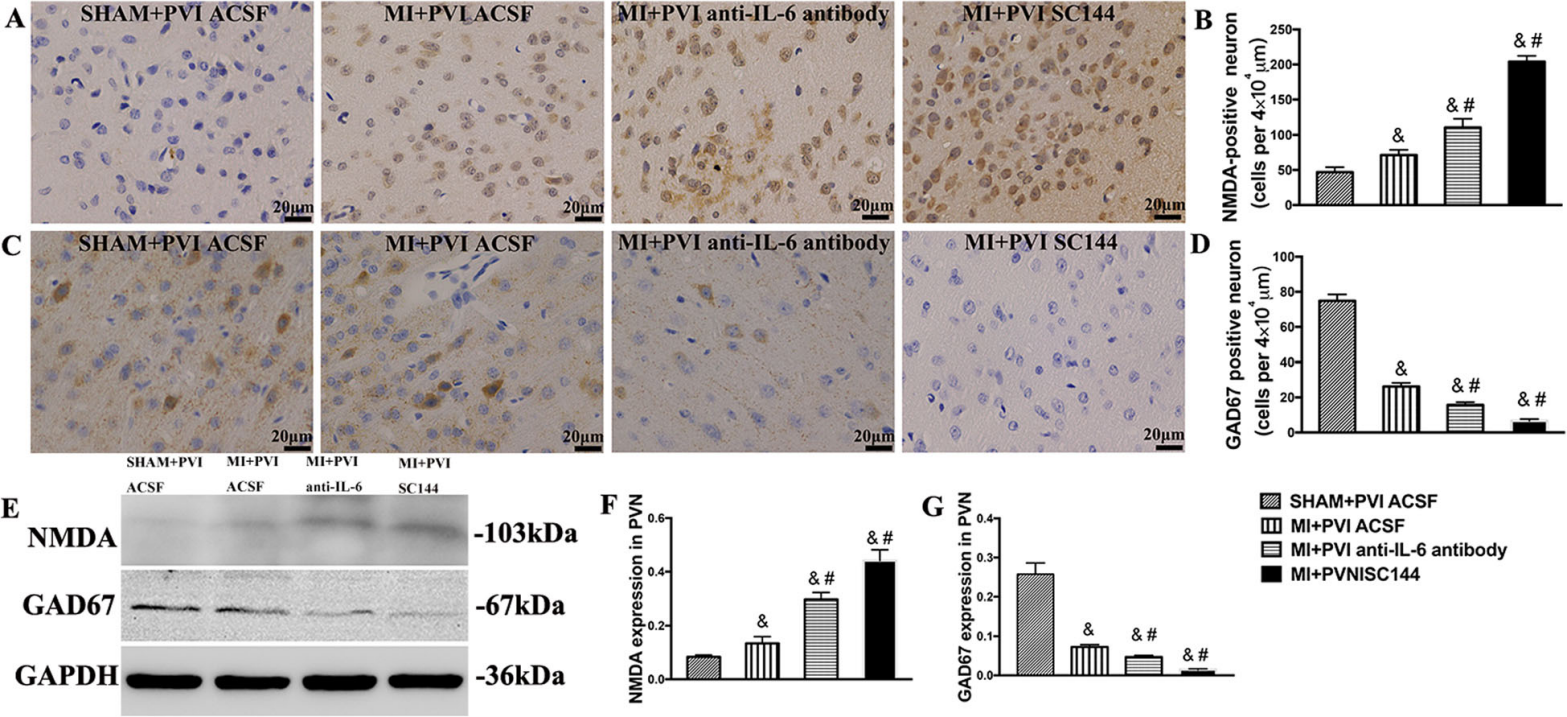
Effects of PVN infusion of an anti-IL-6 antibody or SC144 on NMDA and GAD67 levels within the PVN in MI rats. **a** Representative image of NMDA immunohistochemical staining. **b** Densitometric analysis of NMDA staining. **c** Representative image of GAD67 immunohistochemical staining. **d** Densitometric analysis of GAD67 staining. **e** Representative immunoblot image of NMDA and GAD67 levels. **f** and **g** Densitometric analysis of the protein expression of NMDA and GAD67 (*n* = 4). The values are the means ± SEs. ^&^*p* < 0.05 vs sham rats, ^#^*p* < 0.05 vs MI rats. Abbreviations: PVN, paraventricular nucleus; IL-6, interleukin-6; MI, myocardial infarction

Figure 3a demonstrates the immunohistochemical staining for NMDA receptors in the four groups, and Fig. 3c shows the immunohistochemical staining for GAD67 expression in the four groups. Figure 3b and d display the results of densitometric analysis for NMDA receptors and GAD67, respectively. Figure 3e shows representative immunoblot image of NMDA receptors and GAD67 levels, which demonstrate that MI rats had higher NMDA receptors levels and lower GAD67 levels in the PVN than sham rats (*p* < 0.01). PVN injections of the anti-IL-6 antibody and SC144 promoted an increase of NMDA receptors levels and a decrease of GAD67 levels within the PVNs of MI rats (*p* < 0.05).

### Neurotransmitters in the PVN

**Fig. 4:**
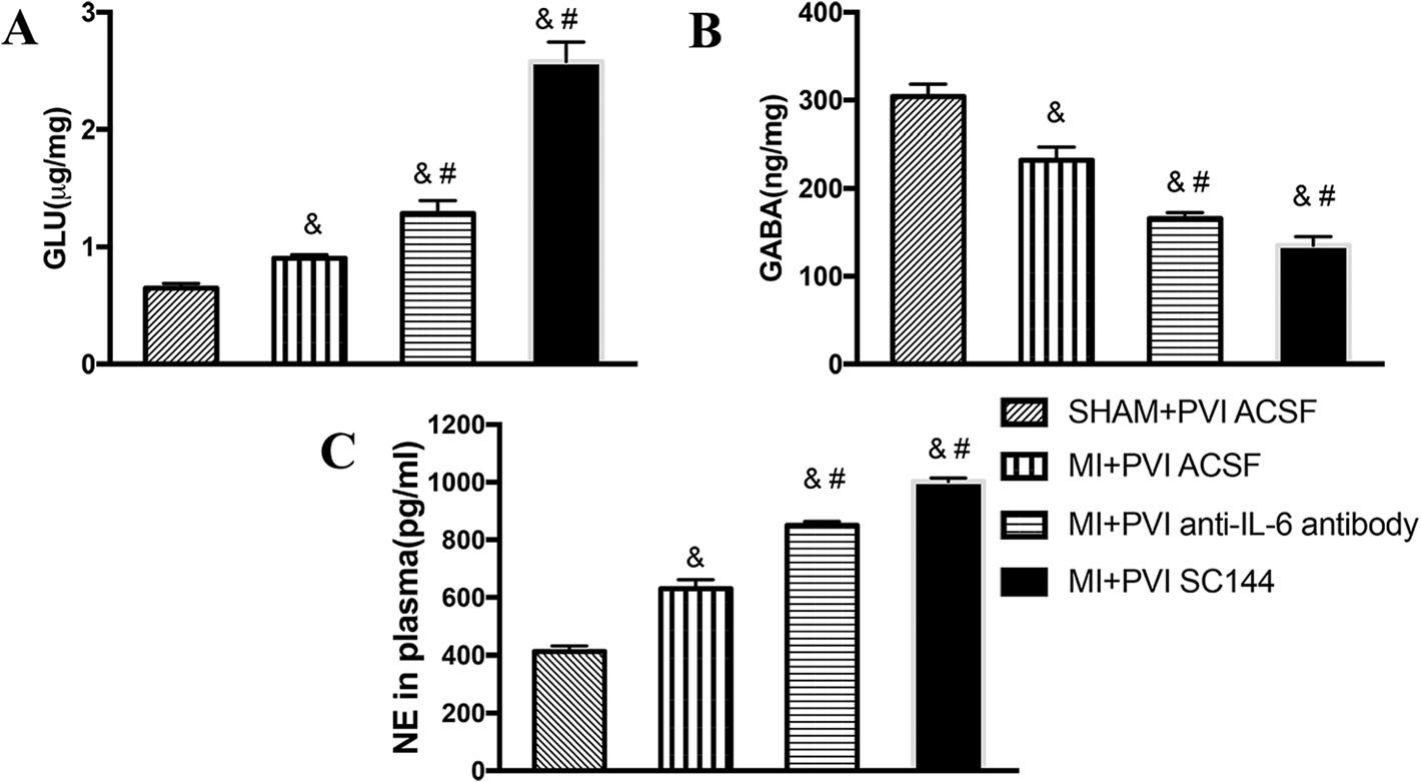
Effects of PVN infusion of an anti-IL-6 antibody or SC144 on PVN glutamate and GABA concentrations in MI rats. **a** Glutamate. **b** GABA. The values are the means ± SEs. **c** Quantitative analysis of plasma NE levels. ^&^*p* < 0.05 vs sham rats, #*p* < 0.05 vs MI rats. Abbreviations: PVN, paraventricular nucleus; IL-6, interleukin-6; MI, myocardial infarction; Gp130: glycoprotein 130; GABA, γ-aminobutyric acid

As shown in Fig. 4a and b, we observed significant differences in the levels of excitatory and inhibitory neurotransmitters in the PVNs of MI and PNI rats compared to those of sham rats. In comparison with sham rats, MI rats had 6-fold higher levels of glutamate (0.64 ± 0.08 vs 0.90 ± 0.09 μg/mg, *p* < 0.05) and 1.3-fold lower levels of GABA in the PVN (304.0 ± 12.0 vs 232.3 ± 9.1 ng/mg, *p* < 0.0001). Furthermore, anti-IL-6 antibody-treated PNI rats and SC144-treated PNI rats had 2-fold and 4-fold higher levels of glutamate (1.28 ± 0.09 μg/mg and 2.57 ± 1.13 μg/mg, respectively) (*p* < 0.0001), and 1.4-fold and 1.7-fold lower levels of GABA (165.9 ± 8.2 ng/mg and 135.2 ± 8.1 ng/mg, respectively) (*p* < 0.0001) in the PVN than MI rats did.

### Plasma humoral factors

To determine the sympathoexcitatory effects of MI and PVN infusion, we measured plasma NE levels in blood using ELISA. Figure 4c shows that, as expected, MI rats showed 1.5-fold higher plasma NE levels than sham rats (412.7 ± 16.5 pg/ml vs 630.5 ± 21.2 pg/ml) (*p* < 0.0001). Additionally, anti-IL-6 antibody-treated PNI rats and SC144-treated PNI rats had about 1.3-fold and 1.5-fold higher levels of plasma NE (850.5 ± 23.2 pg/ml and 1002.0 ± 29.9 pg/ml, respectively) than MI rats did (*p* < 0.0001) (Fig. 4).

## Discussion

The novel finding of the present study is that changes in IL-6 and its downstream molecules Gp130 and STAT3 induce an imbalance between excitatory and inhibitory neurotransmitters and their rate-limiting enzymes in the PVN in MI rats, which contributes to sympathoexcitation and the incidence of VA.

CNS diseases can induce multiple types of arrhythmia, including ventricular tachycardia and VF. Exploring CNS-related ventricular premature contractions is of great significance for clinical work. Thus, the PVN may be a potential target for the prevention and treatment of VA in patients with acute MI. Neuroanatomical studies have shown that the PVN sends direct projections to spinal preganglionic neurons of sympathetic ganglia. Stimulation of cell bodies in the PVN increases blood pressure, heart rate and circulating NE concentrations [31]. Injecting glutamate or a GABA antagonist into the PVN increases renal nerve activity and circulating NE concentrations, suggesting that a sympathoadrenal component to cardiovascular responses is associated with PVN stimulation [32]. Recent research has demonstrated that pathophysiological changes in the PVN are undoubtedly critical to the elevated sympathetic nerve activity in MI. In response to MI, microglia in the PVN become activated and secrete cytokines. In this study, we observed increased expression of IL-6 and activation of Gp130 and STAT3 in PVN neurons of MI rats. Interestingly, while previous studies have demonstrated that other cytokines in the PVN, such as TNF-α and IL-1, play devastating roles, our study indicated that IL-6 in the PVN plays a protective role. When MI rats were treated with an IL-6 antagonist, the sympathetic outflow increased. The role of IL-6 is complicated during inflammation, which contributes to both injury and repair processes. However, the peak in IL-6 expression at 24 h is associated with neuroprotection [13]. Many studies have shown that IL-6 dose-dependently protects neurons against NMDA toxicity. Activation of NMDA receptors can increase sympathetic discharge. In our study, we observed that blocking IL-6 increased glutamate concentrations and elevated NMDA receptor expression in the PVN, whereas it decreased GABA concentrations and reduced GAD67 expression in the PVN.

Moreover, the Gp130-STAT3 pathway plays a key role in this process. Treatment of MI rats with a Gp130 antagonist (SC144) gave rise to the same changes in neurotransmitters in the PVN as treatment with the anti-IL-6 antibody. This effect led to an increase in sympathetic outflow with increased incidence of VA.

Gp130, a common signal-transducing receptor subunit, acts in association with ligand-specific receptors of IL-6. The drugs currently used in the clinic to antagonize IL-6 mostly target IL-6 and IL-6R. However, detrimental side effects, such as bacterial infections, can occur. In this trial, we chose SC144, a novel specific small-molecule inhibitor of Gp130, to block the signal of IL-6, as Gp130 is a new target for IL-6 signalling inhibition [33]. The intracellular signal transduction induced by IL-6 involves the activation of JAK tyrosine kinase family members, leading to the activation of transcription factors of the STAT family. STAT3 is an important element in the JAK-STAT pathway. The phosphorylation of STAT3 at Tyr705 in response to Gp130-stimulating cytokines leads to the formation of STAT3 dimers followed by the translocation of these dimers to the nucleus, where they regulate the transcription of target genes [17].

Increases in cholinergic genes within the stellate ganglion and widespread coexpression of the ChAT protein in TH**+** neurons have been detected in MI rats. The acquisition of cholinergic function requires the expression of the Gp130 cytokine receptor in sympathetic neurons. Removal of Gp130 from sympathetic neurons also prevents local noradrenergic transmission in the left ventricle after acute MI. In this study, Gp130 played the same role in the PVN by transforming glutamate into GABA and inducing an imbalance between excitatory and inhibitory neurotransmitters, thereby further affecting the outflow of sympathetic activity.

## Conclusions

In summary, the present study demonstrates that MI rats have higher concentrations of IL-6, Gp130 and STAT3 in the PVN than sham rats and that the elevations in these molecules contribute to sympathetic nerve inhibition and increased ventricular electrical stability. Our findings provide new insights into the potential treatment of VA in MI rats. Preservation of the IL-6-Gp130-STAT3 pathway in the PVN can reduce the occurrence of VA in the acute phase of MI.

## Limitations

We must consider the effect of the depth of anaesthesia on the autonomic nervous system (ANS). Although we administered anaesthetic according to the weight of each rat, there were individual differences in efficacy. Another limitation of the study is that we elected to diagnose VA from a single-lead electrocardiogram during 24 h of recording. Although the diagnostic accuracy is good in humans, more experiments are needed to confirm the diagnostic accuracy of this method in rats.

## Data Availability

The datasets used and analyzed during the current study are available from the corresponding author on reasonable request.

## Abbreviations

Ach: Acetylcholine
ACSF: Artificial cerebrospinal fluid
ANOVA: Analysis of variance
CNS: Central nervous system
ELISA: Enzyme-linked Immunosorbent assay
GABA: γ-Aminobutyric acid
Gp130: Glycoprotein 130
IL-6: Interleukin-6
JAK: Janus kinase
LSD: Least-significant difference
MI: Myocardial infarction
NE: Norepinephrine
NMDA: N-methyl-D-aspartic acid
PNI: Paraventricular nucleus injection
PVN: Paraventricular nucleus
SDS-PAGE: Sodium salt polyacrylamide gel electrophoresis
SPSS: Statistical product and service solutions
STAT3: Signal transducer and activator of transcription 3
TH: Tyrosine hydroxylase
VA: Ventricular arrhythmia
VF: Ventricular fibrillation
VFT: Ventricular fibrillation threshold
